# Case report: A rare case of meningoencephalitis caused by *Mycobacterium gordonae*

**DOI:** 10.3389/fmed.2024.1416272

**Published:** 2024-10-23

**Authors:** Dezhi Yuan, Xiaomi Ding, Jing Chen, Ying Zhao, Xing Wang, Jie Zhu

**Affiliations:** ^1^Department of Neurology, Chongqing Emergency Medical Center, Chongqing University Central Hospital, Chongqing University, Chongqing, China; ^2^Clinical Medical College, Ya'an Vocational and Technical College, Ya'an, China; ^3^Department of Ultrasonography, Chongqing Emergency Medical Center, Chongqing University Central Hospital, Chongqing University, Chongqing, China

**Keywords:** meningoencephalitis, *Mycobacterium gordonae*, mNGS, double eyelid surgery, case report

## Abstract

Meningoencephalitis, an infectious disease affecting the nervous system, is primarily caused by a variety of pathogens. Non-tuberculous mycobacteria (NTM) have emerged as the leading causative agent of infections worldwide, but central nervous system infections resulting from NTM are infrequent in individuals with functioning immune systems. This case report highlights the diagnosis and treatment of a 26-year-old female patient who developed headaches 2 months post double eyelid surgery and was subsequently diagnosed with NTM meningoencephalitis through metagenomic next-generation sequencing (mNGS) analysis of cerebrospinal fluid. The patient underwent a comprehensive diagnostic and therapeutic protocol, resulting in a positive clinical outcome.

## Introduction

Meningoencephalitis is a neurologic infectious disease caused by a variety of pathogens, including bacteria, viruses, fungi, spirochetes, and rickettsia (1). Non-tuberculous Mycobacteria (NTM), also known as environmental or atypical Mycobacteria, are Mycobacterium species other than Mycobacterium tuberculosis complex or Mycobacterium leprae. While the pathogenicity of NTM is generally lower than that of tuberculous mycobacteria, they have increasingly emerged as a significant cause of infection worldwide due to changes in the infectious disease landscape (2). Infections can manifest in various anatomical locations, such as the lungs, skin, and soft tissues, with the detection frequency of NTM exhibiting an upward trend in recent years (3, 4). Nevertheless, instances of central nervous system infection are infrequent, particularly among immunocompetent individuals (4). Hence, a comprehensive assessment encompassing species diversity, pathogenicity, geographical variations, modes of transmission, and predisposing factors is imperative for the accurate diagnosis of NTM meningoencephalitis. Additionally, the potential role of invasive procedures and implantation of foreign materials in cosmetic surgery, a trend that has gained popularity in recent times (5), should not be underestimated.

## Case presentation

**Figure 1:**
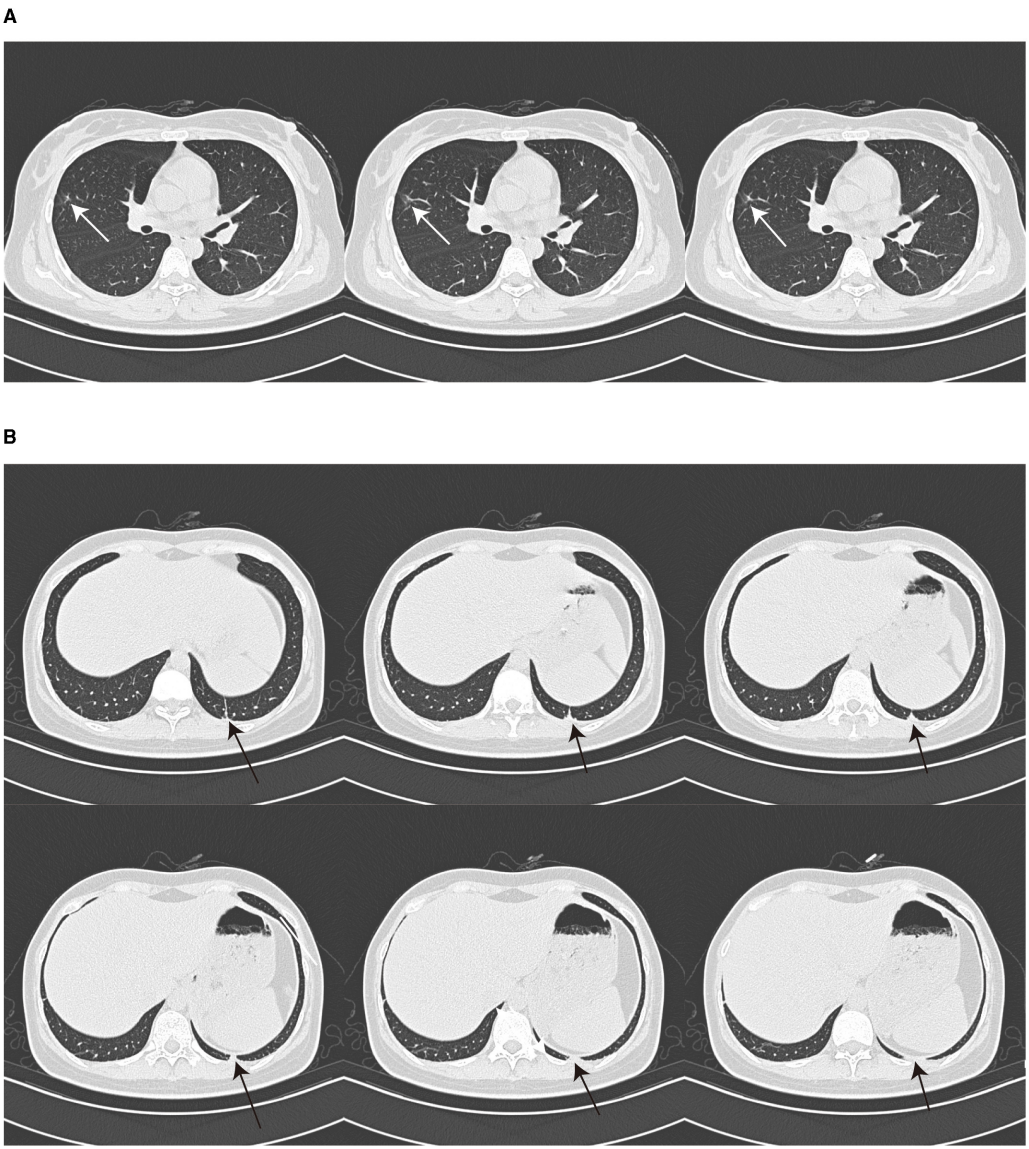
Chest CT image. Chest CT showed a few striated bands in the middle lobe of the right lung [**(A)**, arrow] and segmental atelectasis in the basal segment of the lower lobe of the left lung [**(B)**, arrow].

**Figure 2:**
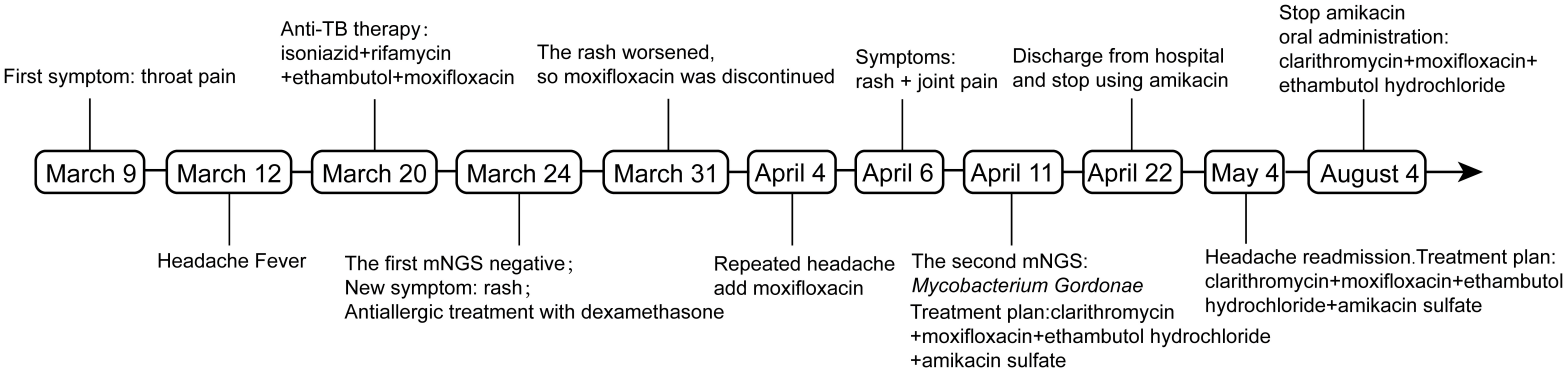
Diagnosis and treatment flow chart.

A 26-year-old Chinese female patient who underwent double eyelid surgery 2 months prior presented with night sweats, loss of appetite, and a subsequent 15 kg weight loss. On 9 March 2021, she reported symptoms of a sore throat, followed by temporal pain and fever (a peak temperature of 38.2°C) 3 days later. Following 4 days of antimicrobial therapy (cefalosporin and levofloxacin) at a community hospital, her sore throat had resolved. However, she had to be referred to our facility for persistent temporal headaches. Upon physical examination, her weight was 53 kg, vital signs were stable, and her cranial nerves, motor system, and sensory system were found to be normal. However, the Babinskis sign and meningeal irritation signs (Neck Stiffness, Kernig's Sign, and Brudzinski's Sign) were positive. The lumbar puncture demonstrated normal intracranial pressure but revealed elevated white blood cell and protein levels in the cerebrospinal fluid (Table 1). The metagenomic next-generation sequencing (mNGS) of the cerebrospinal fluid metagenome (by KingMed Diagnostics Group Co., Ltd.), acid-fast bacillus staining and ink staining were all normal. Chest CT showed a few striated bands within the middle lobe of the right lung and segmental atelectasis in the basal segment of the lower lobe of the left lung (Figures 1A, B). Enhanced CT imaging of the skull was normal. Immunodeficiency syndromes and autoimmune diseases were excluded based on the results of blood specimen testing. Considering her medical history, presenting symptoms of headache and fever, positive Babinskis sign and meningeal irritation signs, as well as the results of cerebrospinal fluid analysis, the possibility of meningoencephalitis (potentially tuberculosis or NTM) should be considered. From March 20th, 2021, four drugs were used (isoniazid, 0.6 g/day through intravenous drip; rifamycin, 0.6 g/day per os; ethambutol 0.75 g/day per os; and moxifloxacin 0.4 g through intravenous drip). The next day, the headache symptoms of the patients were relieved. On the 8th day after treatment, the pressure of cerebral effusion was normal, and the number of white blood cells and monocytes in cerebrospinal fluid decreased. During this period, she developed a rash, and the treatment with dexamethasone did not relieve it. Considering moxifloxacin allergy, the rash improved after suspension, and the remaining three drugs continued to be used according to the treatment guidelines for tuberculous meningoencephalitis. On the 15th day, the patient developed a severe headache again. The initial quadruple anti-tuberculosis treatment is definitely effective, but the regular triple tuberculosis patients have repeated conditions. To control the condition, we restart intravenous injection of moxifloxacin. Subsequently, the patient developed a systemic rash with joint pain. At this time, the second mNGS of cerebrospinal fluid was conducted to identify the pathogen. Upon reanalysis of the original mNGS data, 7 nucleic acid fragments of *Mycobacterium gordonae* were dentified in raw data (Supplementary Table 1). Despite the low number of *M. gordonae* sequences, the possibility of laboratory contamination or detection error was excluded as this bacterial sequence was not found in the cerebrospinal fluid mNGS of other patients from the same batch in the laboratory. In conclusion, she was diagnosed with *M. gordonae* meningoencephalitis. The adjusted treatment plan was clarithromycin 0.75 g/day per os, moxifloxacin 0.8 g/day per os, ethambutol hydrochloride 0.75 g/day per os and amikacin sulfate 0.8 g/day through intravenous drip. And her condition was relieved again. After 1 month of treatment, the patient was discharged from the hospital. Because the patient refused to continue to use amikacin for intravenous injection, she continued to take clarithromycin, moxifloxacin and ethambutol orally after discharge according to China 2020 Guidelines for Diagnosis and Treatment of Non-tuberculous Mycobacterium (6). On the 10th day after discharge, she was readmitted to the hospital with recurrent headaches with nausea. She was no infections were found in her urinary, gynecological, or gastrointestinal examinations. Head MRI showed no abnormality in the brain parenchyma, and chest CT showed no obvious changes. And cerebrospinal fluid pressure is normal, and white blood cells and monocytes in cerebrospinal fluid are increased. We still consider the diagnosis first: NTM meningoencephalitis. Four drugs were used (including clarithromycin 0.75 g/day per os; moxifloxacin 0.8 g/day per os; ethambutol hydrochloride 0.75 g/day per os and amikacin sulfate 0.8 g/day through intravenous drip). After 3 days, the symptoms were relieved. After 3 months of intravenous amikacin infusion, the condition was stabilized and discontinued. After 18 months, she stopped all drug treatment, and her condition was stable without recurrence. The complete treatment process is illustrated in Figure 2.

**Table 1:** CSF profile of the patient.

**Item date**	**Pressure (80–180 mmH_2_O)**	**White cell (0–8 × 10^6^/L)**	**Type of white cell (%)**		**Protein (0.15–0.45 g/L)**	**Glucose (mmol/L)**
			**Monocyte**	**Multinucleated cell**		
8th day headache	180	209	97.6	2.4	2.4	2.27
8th day after treatment	140	24	71	29	-	-
15th day after treatment	130	94	70.9	29.1	0.91	3.05
45th day after treatment	150	50	94.8	5.2	1.12	3.05

## Discussion and conclusion

Initially, it was believed that NTM primarily caused infections in immunocompromised individuals, including those with HIV infection, affecting organs such as the lungs, skin, and soft tissues, with rare transmission through organs (7). Individuals with conditions such as HIV infection, solid organ transplants, corticosteroid use, long-term hemodialysis for kidney failure, diabetes, and malignant tumors are considered at risk for *M. gordonae* infection, and the manifestations of infection are also varied (8, 9). There have been reports of clinically significant infections in immunocompetent individuals, regardless of age, gender, or immune status (9–13). *M. gordonae* infection can be triggered by various factors, the most significant of which are invasive procedures and new surgical interventions (14). Our case study further supports this finding, highlighting the significance of the patient's prior double eyelid surgery.

*M. gordonae* is frequently found in tap water (15, 16) and medical system water (14, 17). There exists a potential risk of NTM infection following invasive eyelid surgery in a hospital setting. In this case the patient presented with night sweats, loss of appetite, weight loss, followed by sore throat and temporal pain after cosmetic eyelid surgery and was finally diagnosed with intracranial infection caused by *M. gordonae*. The association between this type of infection and double eyelid surgery cannot be disregarded. It is well-established that the facial vein and ocular vein play a crucial role in draining venous blood from the facial region, eyeball, and ocular appendages. The ocular vein connects with the facial vein anteriorly and communicates with intracranial veins posteriorly. Furthermore, the ocular vein lacks valves to regulate blood flow direction, potentially allowing facial infections to spread intracranially through the ocular vein (18). The invasive nature of double eyelid surgery may increase the risk of infectious agents entering the surgical site (19). Our case reports suggest a need for reevaluation of the potential severity of surgical complications associated with this common medical cosmetic procedure.

Prior studies have indicated that NTM infections in immunocompetent individuals, initially presenting as meningoencephalitis, exhibit a rapid progression and pose challenges in diagnosis, often resulting in mortality within a 2-week timeframe and necessitating post-mortem confirmation. Conversely, survivors typically display localized lesions, such as intracranial encapsulated abscesses, with diagnosis typically confirmed through post-surgical pathological examination (20). Currently, routine microbial culture remains the diagnostic tool for NTM infections. However, some NTM cultures necessitate the utilization of specialized media, the adherence to incubation temperatures, or the extension of the incubation period. And the presence of a lower pathogenic microorganism load in cerebrospinal fluid, posing challenges to clinical diagnosis due to the limited sensitivity of traditional diagnostic methods such as cultures and smears. In contrast, metagenomic next-generation sequencing (mNGS) offers improved sensitivity and specificity in detecting pathogenic bacteria compared to culture methods (21, 22). mNGS is capable of identifying pathogenic microorganisms in various clinical specimens, including cerebrospinal fluid (23–25). Hence, mNGS offers direct detection of seven nucleic acid fragments of *M. gordonae*, presenting significant implications for early diagnosis. The concurrent utilization of mNGS, cultures, and smears, along with meticulous comparison of results obtained from the same set of samples, may represent an optimal approach for attaining expedited and precise diagnostic outcomes.

Four efficacious anti-*M. gordonae* medications were selected for the treatment regimen: clarithromycin, moxifloxacin, ethambutol hydrochloride, and amikacin. Clarithromycin (26, 27), a macrolide antibiotic, exerts its bacteriostatic effects by inhibiting protein synthesis through the obstruction of the 50S ribosomal subunit. Over the past two decades, clarithromycin has been the cornerstone of treatment for NTM infections due to its broad-spectrum efficacy against various NTM species, including *M. gordonae*. Furthermore, clarithromycin is capable of crossing the blood-brain barrier (BBB) in cases of meningoencephalitis. The recommended dosage for adults weighing <50 kg is 500–750 mg per day, whereas adults weighing 50 kg or more should receive 750–1,000 mg per day, with a maximum daily dose of 1,000 mg. Moxifloxacin (26–28) functions by inhibiting and preventing the replication and transcription of bacterial DNA, thereby exerting a sterilizing effect on the bacteria. It exhibits a more pronounced efficacy against slow-growing NTM, such as *M. gordonae*, while also demonstrating antibacterial activity against fast-growing NTM. Additionally, moxifloxacin has been shown to possess favorable permeability across the BBB. The established dosage regimen for adult's ranges from 400 to 800 mg per day, administered either orally or intravenously once daily. In this case, the patient exhibited a skin rash and joint pain following intravenous administration, which are recognized as common allergy and adverse effects. Nonetheless, these symptoms resolved upon transitioning to oral administration, suggesting that the route of administration may impact the manifestation of drug side effects. The concomitant use of moxifloxacin with other anti-NTM agents has demonstrated an enhancement in the drug's efficacy. It must be noted that moxifloxacin is associated with cardiotoxicity, specifically the prolongation of the Q-T interval. Ethambutol (25, 29) is the most commonly utilized and essential pharmaceutical agent in the treatment of NTM disease. It functions by inhibiting the synthesis of the cell wall of Mycobacterium and demonstrates antibacterial activity against *M. gordonae*. The recommended dosage for adults is 15–25 mg/kg/day, with a maximum daily limit of 1,250 mg. Amikacin (26, 30) exhibits a more pronounced bactericidal effect against *M. gordonae*, with an adult dosage of 15–20 mg/kg/day and a maximum daily limit of 1.0 g. The administration of this treatment is conducted through either intramuscular injection or intravenous infusion. However, the cessation of amikacin during the treatment course led to an extended duration of therapy, potentially affecting the ultimate therapeutic outcome. Subsequent to the second intervention, the patient expressed a commitment to adhere to the prescribed treatment regimen, which includes combination pharmacotherapy and prolonged intravenous infusions. The treatment protocol for this case indicates the necessity of considering several critical factors: the selection of appropriate therapeutic agents must be guided by the specific type of infectious agent, the pattern of drug resistance, and the site of infection. Notably, clarithromycin, ethambutol, and amikacin possess the capability to traverse the BBB and exert antimicrobial effects within the central nervous system, particularly when inflammation has compromised the integrity of the BBB. Furthermore, rigorous safety monitoring and management and treatment management are essential for all patients undergoing treatment for NTM disease. Currently, there is an absence of standardized treatment protocols for NTM infections affecting the central nervous system. This case study could provide valuable insights for the diagnosis and management of such patients in the future.

This case represents the initial documentation of *M. gordonae* meningoencephalitis arising from eyelid surgery, and notably, the first instance in a non-immunocompromised individual where the condition presented as meningoencephalitis and was successfully treated. Meningoencephalitis caused by NTM infection is characterized by its swift and aggressive progression. Utilization of mNGS can aid in the prompt identification of NTM infection, yet it is imperative to meticulously compare laboratory parameters and test outcomes from a consistent set of samples to arrive at a comprehensive assessment. Furthermore, timely diagnosis and appropriate, comprehensive, consistent, and sustained pharmacological treatment yield favorable clinical results. Additionally, the first documented cases of NTM meningoencephalitis linked to medical cosmetic surgery highlight the emergence of novel disease risks, particularly in light of the growing prevalence of medical cosmetic surgery.

## Data Availability

The original contributions presented in the study are included in the article/Supplementary material, further inquiries can be directed to the corresponding author.
