# Supplementary material for: Case report: A rare case of meningoencephalitis caused by Mycobacterium gordonae
Source: Front Med (Lausanne). 2024 Oct 23;11:1416272. doi: 10.3389/fmed.2024.1416272 (PMC11541715; doi:10.3389/fmed.2024.1416272)
Supplement: Supplementary file 1 [file Table_1.DOCX]

Supplementary Table 1 Results of metagenomic sequencing

| Latin | Coverage | CovRate | Depth | Latin_mrn | Latin_smrn | GenusLatin | G_MRN | G_SMRN | Depth_Ratio | Shannon_index |
| --- | --- | --- | --- | --- | --- | --- | --- | --- | --- | --- |
| Mycobacterium_gordonae | 544/6929818 | 0.0079 | 1 | 22 | 7 | Mycobacterium | 155 | 19 | 1 | 0.98 |
| Mycolicibacterium_tusciae | 500/6163570 | 0.0081 | 1 | 12 | 6 | Mycolicibacterium | 392 | 143 | 1 | 0.98 |
| Mycobacterium_europaeum | 164/6152643 | 0.0027 | 1 | 4 | 2 | Mycobacterium | 155 | 19 | 1 | 0.51 |
| Mycobacterium_asiaticum | 126/5890313 | 0.0021 | 1 | 6 | 1 | Mycobacterium | 155 | 19 | - | - |
| Mycobacterium_sherrisii | 122/5688440 | 0.0021 | 1 | 3 | 1 | Mycobacterium | 155 | 19 | - | - |
| Mycobacterium_sp._JLS | 86/6048425 | 0.0014 | 1 | 2 | 1 | Mycobacterium | 155 | 19 | - | - |
| Mycolicibacterium_moriokaense | 119/7124508 | 0.0017 | 1 | 5 | 0 | Mycolicibacterium | 392 | 143 | - | - |
| Mycolicibacterium_rutilum | 248/5987931 | 0.0041 | 1 | 6 | 0 | Mycolicibacterium | 392 | 143 | 1 | 0.92 |
| Mycobacterium_alsense | 43/5659948 | 0.0008 | 1 | 1 | 0 | Mycobacterium | 155 | 19 | - | - |
| Mycobacterium_arosiense | 86/5984846 | 0.0014 | 1 | 2 | 0 | Mycobacterium | 155 | 19 | - | - |
| Mycobacterium_avium | 129/5892371 | 0.0022 | 1 | 3 | 0 | Mycobacterium | 155 | 19 | - | - |
| Mycobacterium_chimaera | 121/6608419 | 0.0018 | 1 | 3 | 0 | Mycobacterium | 155 | 19 | - | - |
| Mycobacterium_colombiense | 80/5947639 | 0.0013 | 1 | 2 | 0 | Mycobacterium | 155 | 19 | - | - |
| Mycobacterium_conspicuum | 86/6201524 | 0.0014 | 1 | 2 | 0 | Mycobacterium | 155 | 19 | - | - |
| Mycobacterium_fragae | 40/4731387 | 0.0008 | 1 | 1 | 0 | Mycobacterium | 155 | 19 | - | - |
| Mycobacterium_gastri | 164/6011133 | 0.0027 | 1 | 4 | 0 | Mycobacterium | 155 | 19 | 1 | 0.95 |
| Mycobacterium_genavense | 85/4936311 | 0.0017 | 1 | 2 | 0 | Mycobacterium | 155 | 19 | - | - |
| Mycobacterium_haemophilum | 115/4370158 | 0.0026 | 1 | 3 | 0 | Mycobacterium | 155 | 19 | - | - |
| Mycobacterium_heckeshornense | 43/5012073 | 0.0009 | 1 | 1 | 0 | Mycobacterium | 155 | 19 | - | - |
| Mycobacterium_heidelbergense | 43/5000195 | 0.0009 | 1 | 1 | 0 | Mycobacterium | 155 | 19 | - | - |
| Mycobacterium_interjectum | 166/5904856 | 0.0028 | 1 | 4 | 0 | Mycobacterium | 155 | 19 | 1 | 0.95 |
| Mycobacterium_intermedium | 123/6857760 | 0.0018 | 1 | 3 | 0 | Mycobacterium | 155 | 19 | - | - |
| Mycobacterium_intracellulare | 35/6332156 | 0.0006 | 1 | 1 | 0 | Mycobacterium | 155 | 19 | - | - |
| Mycobacterium_kansasii | 158/6896682 | 0.0023 | 1 | 4 | 0 | Mycobacterium | 155 | 19 | 1 | 0.51 |
| Mycobacterium_kubicae | 129/5833718 | 0.0022 | 1 | 3 | 0 | Mycobacterium | 155 | 19 | - | - |
| Mycobacterium_kyorinense | 125/4832982 | 0.0026 | 1 | 5 | 0 | Mycobacterium | 155 | 19 | - | - |
| Mycobacterium_lacus | 43/4907148 | 0.0009 | 1 | 1 | 0 | Mycobacterium | 155 | 19 | - | - |
| Mycobacterium_lentiflavum | 43/6818547 | 0.0006 | 1 | 1 | 0 | Mycobacterium | 155 | 19 | - | - |
| Mycobacterium_malmoense | 405/5667851 | 0.0071 | 1.25 | 16 | 0 | Mycobacterium | 155 | 19 | 0.8 | 0.52 |
| Mycobacterium_mantenii | 121/5838798 | 0.0021 | 1 | 3 | 0 | Mycobacterium | 155 | 19 | - | - |
| Mycobacterium_marinum | 43/6636827 | 0.0006 | 1 | 1 | 0 | Mycobacterium | 155 | 19 | - | - |
| Mycobacterium_nebraskense | 35/6729057 | 0.0005 | 1 | 1 | 0 | Mycobacterium | 155 | 19 | - | - |
| Mycobacterium_noviomagense | 333/4742000 | 0.007 | 1.16 | 9 | 0 | Mycobacterium | 155 | 19 | 0.86 | 0.32 |
| Mycobacterium_palustre | 115/6039092 | 0.0019 | 1 | 3 | 0 | Mycobacterium | 155 | 19 | - | - |
| Mycobacterium_paraffinicum | 172/6476152 | 0.0027 | 1 | 4 | 0 | Mycobacterium | 155 | 19 | 1 | 0.63 |
| Mycobacterium_paraintracellulare | 36/5501090 | 0.0007 | 1 | 1 | 0 | Mycobacterium | 155 | 19 | - | - |
| Mycobacterium_parascrofulaceum | 43/6565401 | 0.0007 | 1 | 1 | 0 | Mycobacterium | 155 | 19 | - | - |
| Mycobacterium_parmense | 129/5892640 | 0.0022 | 1 | 3 | 0 | Mycobacterium | 155 | 19 | - | - |
| Mycobacterium_riyadhense | 43/6272470 | 0.0007 | 1 | 1 | 0 | Mycobacterium | 155 | 19 | - | - |
| Mycobacterium_saskatchewanense | 119/5931795 | 0.002 | 1 | 3 | 0 | Mycobacterium | 155 | 19 | - | - |
| Mycobacterium_scrofulaceum | 160/6172103 | 0.0026 | 1 | 4 | 0 | Mycobacterium | 155 | 19 | 1 | 0.95 |
| Mycobacterium_shimoidei | 168/4842681 | 0.0035 | 1 | 4 | 0 | Mycobacterium | 155 | 19 | 1 | 0.95 |
| Mycobacterium_shinjukuense | 113/4411686 | 0.0026 | 1 | 3 | 0 | Mycobacterium | 155 | 19 | - | - |
| Mycobacterium_simiae | 80/6687309 | 0.0012 | 1 | 2 | 0 | Mycobacterium | 155 | 19 | - | - |
| Mycobacterium_sp._KMS | 43/5737227 | 0.0007 | 1 | 1 | 0 | Mycobacterium | 155 | 19 | - | - |
| Mycobacterium_sp._MCS | 167/5920533 | 0.0028 | 1 | 4 | 0 | Mycobacterium | 155 | 19 | 1 | 0.63 |
| Mycobacterium_sp._MOTT36Y | 43/5613626 | 0.0008 | 1 | 1 | 0 | Mycobacterium | 155 | 19 | - | - |
| Mycobacterium_szulgai | 123/6674429 | 0.0018 | 1 | 3 | 0 | Mycobacterium | 155 | 19 | - | - |
| Mycobacterium_triplex | 75/6382870 | 0.0012 | 1 | 2 | 0 | Mycobacterium | 155 | 19 | - | - |

Supplementary Table 1: Results of the second cerebrospinal fluid metagenomic sequencing
